# Haemophagocytic lymphohistiocytosis and abdominal compartment syndrome in acute pancreatitis

**DOI:** 10.1093/jscr/rjaf813

**Published:** 2025-10-14

**Authors:** Nishka Tapaswi, Hasib Ahmadzai, Alec J Hope, Oliver Fisher, Linda Y Zhang

**Affiliations:** Department of Gastroenterology and Hepatology, St George Public Hospital, Gray St, Kogarah, NSW 2217, Australia; University of New South Wales, High St, Kensington, NSW 2052, Australia; Department of Gastroenterology and Hepatology, St George Public Hospital, Gray St, Kogarah, NSW 2217, Australia; Department of Gastroenterology and Hepatology, St George Public Hospital, Gray St, Kogarah, NSW 2217, Australia; Department of Upper Gastrointestinal Surgery, St George Public Hospital, Gray St, Kogarah, NSW 2217, Australia; Department of Gastroenterology and Hepatology, St George Public Hospital, Gray St, Kogarah, NSW 2217, Australia

**Keywords:** pancreatitis, acute necrotising pancreatitis, hemophagocytic lymphohistiocytosis, intra-abdominal hypertension, abdominal compartment syndrome

## Abstract

Haemophagocytic lymphohistiocytosis (HLH) is a rare and often fatal syndrome that may be primary or secondary. This is a case of HLH associated with necrotizing pancreatitis, complicated by abdominal compartment syndrome. It highlights the need for high degree of suspicion for diagnosis in surgical patients, the complexity of managing the sequalae of HLH, and the importance of early treatment.

## Introduction

Haemophagocytic lymphohistiocytosis (HLH) is a rare syndrome of uncontrolled haemophagocytosis and cytokine overproduction that may be primary, or secondary to malignancy, infection, and autoimmunity [1]. There are currently six previously reported cases linking acute pancreatitis and HLH; in three cases no other secondary cause was identified, and no case was associated with haemorrhagic pancreatitis [2–7]. We describe a rare case of HLH associated with necrotising pancreatitis and abdominal compartment syndrome.

## Case report

**Figure 1:**
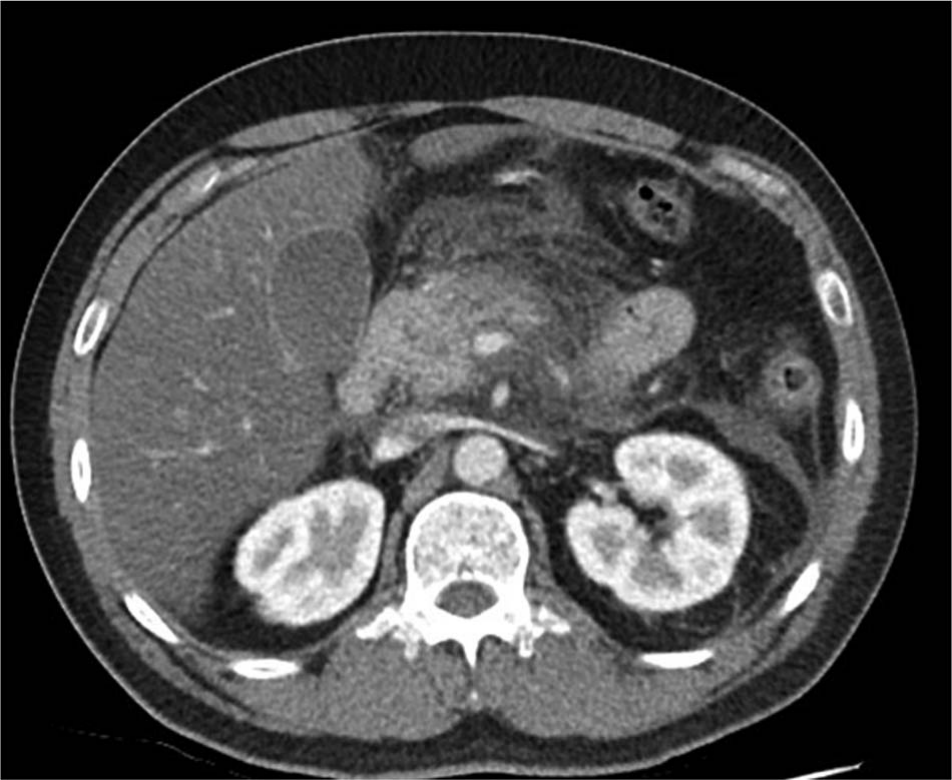
Axial CT image demonstrating body and tail pancreatitis with fat stranding.

**Figure 2:**
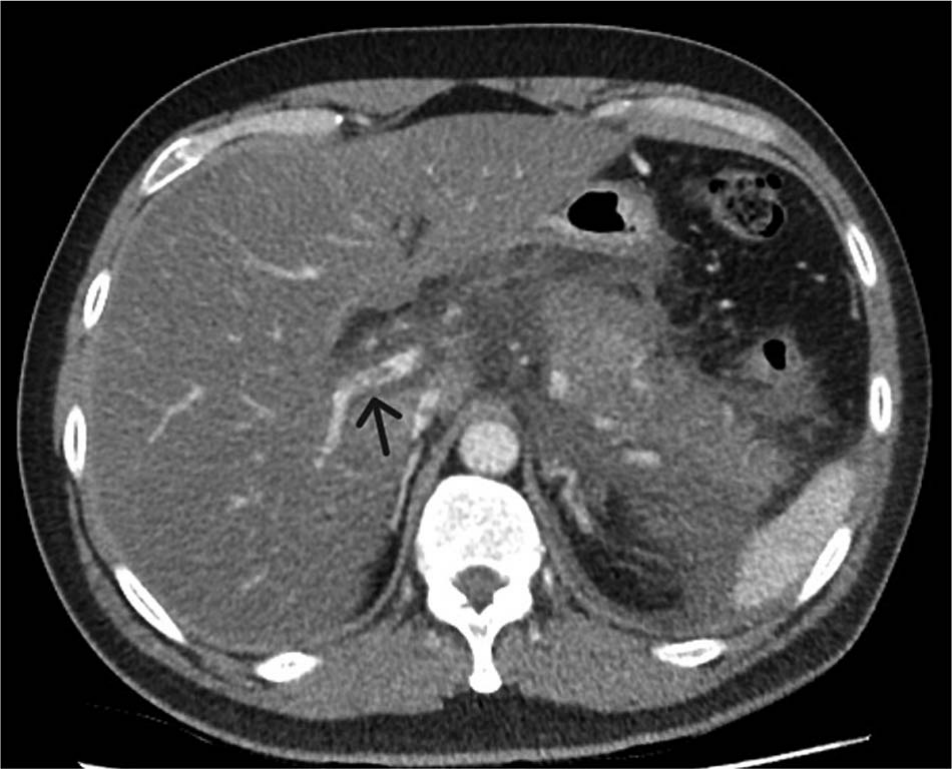
Axial CT image demonstrating non-occlusive portal vein thrombus (depicted by arrow).

**Figure 3:**
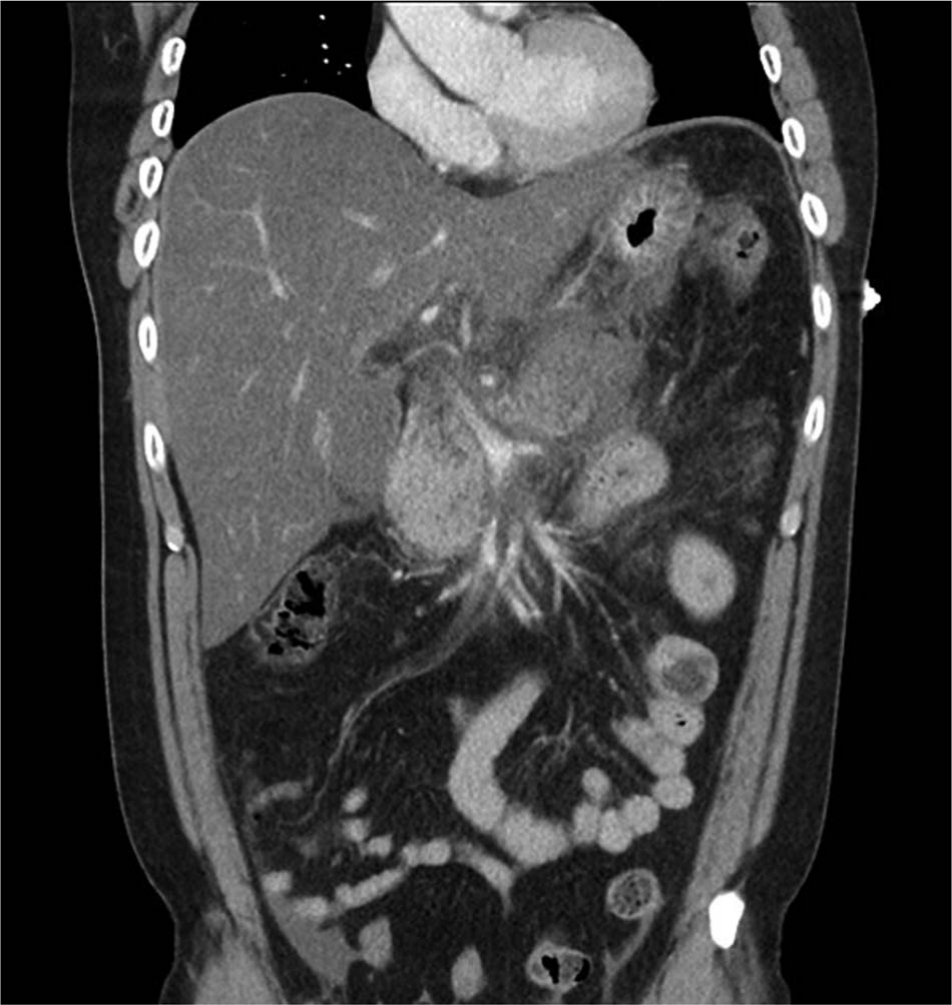
Coronal CT image demonstrating body and tail pancreatitis with potential early signs of necrosis.

A 39-year-old male presented with one week of worsening epigastric pain radiating to the back, with vomiting, jaundice, and dark urine. His past medical history included depression and alcohol-related liver disease without cirrhosis. On admission he was afebrile, had a soft abdomen with epigastric tenderness worse on palpation, tachycardic, and normotensive. Initial investigations showed a lipase of 2600 U/L, C-reactive protein 238 mg/L, lactate 3.4 mmol/L, and bilirubin 100 μmol/L (Table 1). Abdomen and pelvic computer tomography (CT) demonstrated body and tail pancreatitis, fat stranding, portal vein thrombus, and early necrosis signs, with no choledocholithiasis (Figs 1–3).

**Table 1:** Biochemical markers on Day 1 and Day 34 of admission

**Biochemical marker**	**Standard range**	**Day 1**	**Day 34**
Creatinine (μmol/L)	50–100	140	117
eGFR (ml/min/1.73m^2^)	>90	54	67
Sodium (mmol/L)	135–145	129	127
Potassium (mmol/L)	3.5–5.2	3.7	5.9
Bilirubin (μmol/L)	0–20	100	487
ALP (U/L)	50–220	169	
GGT (U/L)	5–50	569	
ALT (U/L)	<51	226	85
AST (U/L)	<36		62
White cell count (x10^9^/L)	3.5–11.0	10.10	1.70
Haemoglobin (g/L)	130–180	185	76
Platelets (x10^9^/L)	150–450	223	51
Triglycerides (mmol/L)	<2.0	9.2	
Iron level (μmol/L)	8.1–32.6	3.5	
Transferrin (g/L)	1.8–3.5	0.8	
Ferritin (μg/L)	20–300	3184	10 281

Abbreviations: eGFR, estimated glomerular filtration rate; ALP, alkaline phosphatase; GGT, gamma-glutamyltransferase; ALT, alanine aminotransferase; AST, aspartate aminotransferase.

He was diagnosed with severe acute pancreatitis secondary to alcohol intake. Upon Intensive Care Unit admission, he was fluid resuscitated with intravenous crystalloid, and the portal vein thrombus was treated with intravenous heparin infusion. Magnetic resonance cholangiopancreatography showed peri-pancreatic fluid, formation of intra- and retro-peritoneal collections, and subtle common bile duct (CBD) narrowing. Deteriorating renal function necessitated continuous renal replacement therapy.

Given his significant clinical deterioration with persistent jaundice and fevers, a multidisciplinary decision was made to proceed with endoscopic retrograde cholangiopancreaticography (ERCP) on Day 6, despite no evidence of obstruction or duct dilation. This demonstrated indeterminate CBD narrowing without an obvious stricture or filling defect. A 10Fr × 7 cm plastic biliary stent was placed in the CBD, and a nasojejunal feeding tube was inserted. Post-anaesthesia, he was intubated due to agitation and hypoxia. There was no subsequent decrease in bilirubin.

CT abdomen one week after admission demonstrated features of necrotizing pancreatitis and increased retroperitoneal fluid. He was commenced on piperacillin and tazobactam. On Day 12, he became haemodynamically unstable with a drop in haemoglobin to 66 g/L, requiring vasopressor support and blood transfusion. A CT mesenteric angiogram demonstrated active retroperitoneal haemorrhage from the right psoas muscle and lumbar artery that was embolized with interventional radiology.

Despite two embolization attempts, he developed worsening elevated intra-abdominal pressures (21 cm H_2_O) with abdominal distension and haemodynamic instability. On Day 15, urinary catheterization and medical treatment of ileus and faecal loading failed to reduce intra-abdominal pressures. A decompressive laparotomy was performed as ventilatory parameters were deteriorating. This demonstrated minimal free fluid, with mesenteric and omental inflammation, and tethered but healthy bowel. The abdomen was left open with an AbThera™ dressing.

He continued to have worsening haemodynamic parameters and a relook laparotomy the following day showed protrusion of retroperitoneal necrotic material in the anterior abdominal compartment. A washout of clot and necrotic tissue was performed, and the lesser sac was packed to tamponade ooze. He required further laparotomies due to bleeding and septic complications. Given persistently elevated intra-abdominal pressures anticipated on closure of the abdomen, he underwent CT-guided botulinum toxin injection (300 IU) into the abdominal wall musculature to facilitate staged closure. His bilirubin continued to rise, and he underwent repeat ERCP for stent exchange. This found haemobilia from the left-sided biliary segments.

**Figure 4:**
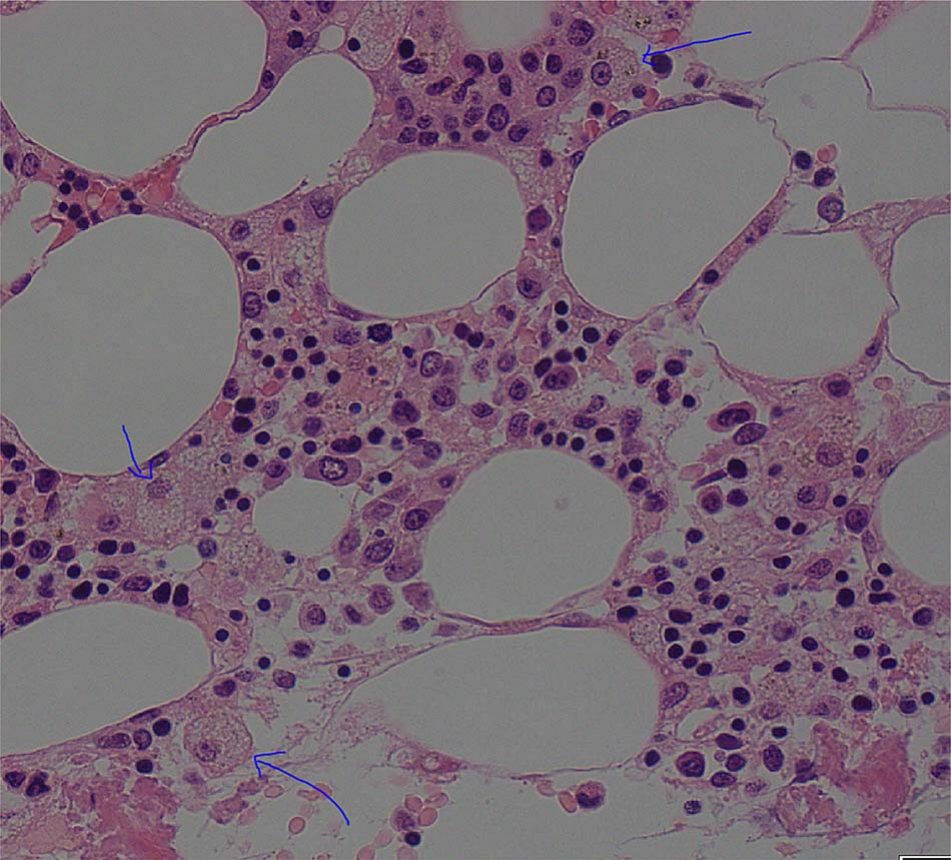
Low power view (100× magnification) of bone marrow aspirate smear with haematoxylin and eosin staining with multiple histiocytes demonstrated by blue arrows.

**Figure 5:**
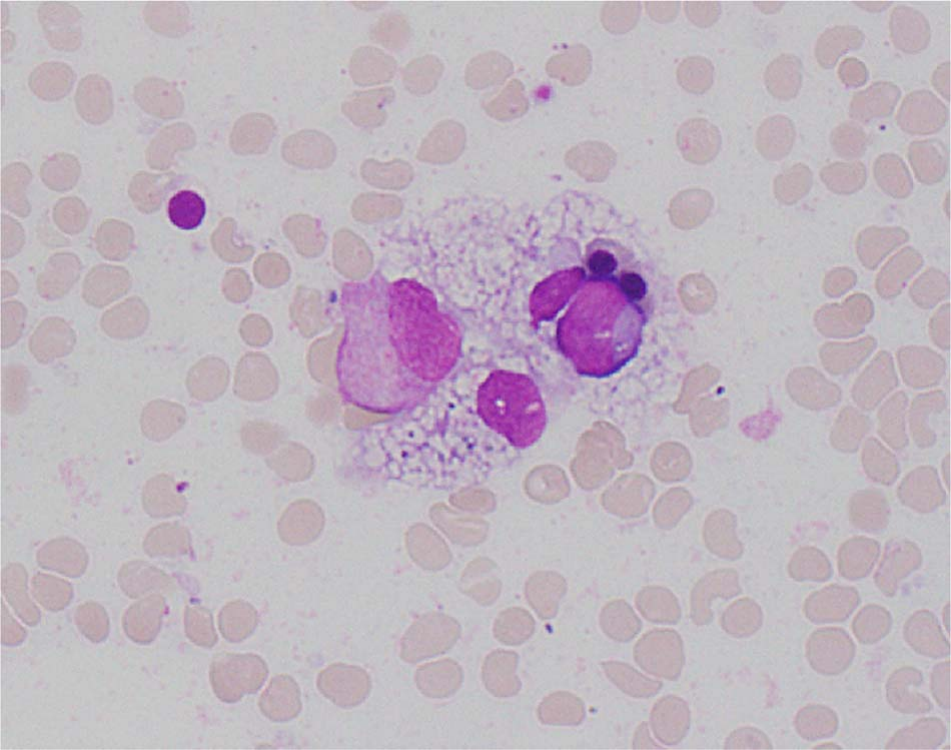
High power view (1000× magnification) of bone marrow aspirate smear with haematoxylin and eosin staining demonstrating haemophagocytosis by histiocyte (depicted in centre).

On Day 34, new pancytopenia (white cell count of 1.7 × 10^9^/L, Hb 76 g/L, platelets 61 × 10^9^/L; Table 1), along with persistent fevers and hyperbilirubinaemia, suggested a HLH diagnosis, not previously considered as there were many causes for the patient to be febrile. Haematology recommended a bone marrow biopsy that demonstrated increased histiocytes with haemophagocytosis (Figs 4–5). HLH likelihood was >99% [8–9] (H-score = 258) which was suggestive of HLH secondary to severe necrotizing pancreatitis and intra-abdominal infection.

Haematology and infectious disease weighed up concerns of worsening sepsis against immunosuppression for HLH treatment. His antibiotics were switched to Aztreonam, and he received 15 mg dexamethasone daily, and two 80 g doses of intravenous immonoglobulin. This resulted in moderate improvement in fevers and noradrenaline requirement on Day 35.

Operative swabs from a laparotomy on Day 36 grew *Citrobacter*, *Enterococcus faecium*, and *Candida*, with blood cultures on Day 37 growing *E. faecium*. During a laparotomy the next day, the patient became haemodynamically unstable and needed angioembolization of the proximal splenic artery. He received eight units of packed red blood cells and blood factor replacement.

Further intraabdominal haemorrhage resulted in worsening abdominal distension and acidosis, necessitating subsequent laparotomy and embolization attempts. After a family meeting, a decision was made to transition to comfort care given the low probability of recovery, and he passed away, 7 weeks after his admission.

## Discussion

Early recognition and treatment of HLH in surgical patients is crucial to prevent death. This requires a high degree of suspicion when criteria for the H-score for secondary HLH are met, such as cytopaenias, hypertriglyceridaemia, and hyperferritinaemia [9]. A fever in the first and second weeks onwards of pancreatitis are generally associated with inflammation and sepsis, respectively [4]. Thus, while distinguishing sepsis from HLH is clinically challenging due to overlapping inflammatory features, recognizing the aforementioned biochemical markers is crucial, as they increase the likelihood of either sole HLH or a mixed HLH and sepsis picture [10]. Our patient also underwent multiple ERCPs to manage his hyperbilirubinaemia, which is not a diagnostic feature of HLH, but has been shown to be a poor prognostic marker, increasing mortality particularly with concomitant viral infection [11].

Finally, abdominal compartment syndrome is a complication seen in 40%–50% of severe acute pancreatitis [12]. In our patient, HLH associated coagulopathy likely compounds this risk. As per World Society of the Abdominal Compartment Syndrome guidelines, abdominal decompression was performed due to the consistently elevated abdominal pressures despite medical management [13]. These attempts were likely unsuccessful as the underlying coagulopathy had not been addressed [14]. This reiterates the importance of early detection of HLH and a multidisciplinary surgical and medical management approach, including early haematology assessment with consideration of bone marrow biopsy.
